
JOURNAL INFORMATION
==============================
NLM Title Abbreviation: Wirtschaftsdienst
Journal ID: Wirtschaftsdienst
NLM Journal ID: 101086612

Wirtschaftsdienst (Hamburg, Germany : 1949)

ISSN: 0043-6275

ARTICLE INFORMATION
==============================
PMCID: PMC8598271
PMID: 34812208
DOI: 10.1007/s10273-021-3044-9
Article ID: 3044
Article version: 1
Subjects: Zeitgespräch

Globale Gesundheitssicherheit als moralische und wirtschaftliche Chance

Glassman Amanda aglassman@cgdev.org
1
Wolff Guntram B. guntram.wolff@bruegel.org
2
1 grid.466498.1 0000 0001 2295 2115 Director Global Health Policy, Center for Global Development, 2055 L Street NW. Fifth Floor, 20036 Washington, D.C., USA
2 grid.432713.0 Bruegel, Rue de la Charité 33, 1210 Brussels, Belgium
Electronic publication date: 2021 Nov 18
Print publication date: 2021
Volume: 101
Issue: 11
First page: 858
Last page: 861
Copyright: © Der/die Autor:in 2021
License: Open Access: Dieser Artikel wird unter der Creative Commons Namensnennung 4.0 International Lizenz veröffentlicht (https://creativecommons.org/licenses/by/4.0/deed.de).
Open Access wird durch die ZBW — Leibniz-Informationszentrum Wirtschaft gefördert.
License URL: https://creativecommons.org/licenses/by/4.0/

issue-copyright-statement: © The Author(s) 2021

==============================
Amanda Glassman ist Executive Vice President des Center for Global Development, Chief Executive Officer der CGD Europe sowie Senior Fellow am Center for Global Development in Washington.

Dr. Guntram B. Wolff ist Direktor des Bruegel Institut in Brüssel. Er war Mitglied im G20 High Level Independent Panel on Financing Pandemic Preparedness, Prevention and Response (HLIP).

Die COVID-19-Pandemie hat die Fragilität der Architektur globaler Gesundheitssysteme im Katastrophenfall enthüllt. Die verhaltene und schleppende Bereitstellung internationaler Finanzierung hat weltweit zu vielen Infektionen und Todesfällen beigetragen. Neue Pandemiewellen drohen jetzt den Wiederaufschwung zu gefährden. Gerade die Bundesrepublik Deutschland hat ein großes Interesse daran, die Pandemie nicht nur in Deutschland, sondern weltweit in den Griff zu bekommen und zukünftige Pandemien möglichst zu verhindern. Deutschlands enge wirtschaftliche Verflechtung mit der Welt bedeutet, dass Störungen in Lieferketten oder Nachfrageeinbrüche während Pandemien auch aus reinem deutschen Eigeninteresse äußerst negativ zu bewerten sind. Es gilt deshalb auch für die neue deutsche Bundesregierung, eine geeignete Strategie für die globale Gesundheitssicherheit zu verfolgen – und dies auch als moralische und wirtschaftliche Chance zu begreifen.

Wir nähern uns dem Ende des zweiten Jahres, in dem wir mit dem sich ausbreitenden und mutierenden Coronavirus leben. Europa und Deutschland haben sowohl eine moralische als auch eine wirtschaftliche Chance, ihr Engagement für die Weltgesundheit weiter zu verstärken. Deutschland kann dabei seine wachsende Präsenz im Bereich der globalen Gesundheit und seine neue globale Gesundheitsstrategie nutzen, um weltweit einen gerechteren Zugang zu Impfstoffen und Behandlungen gegen Corona zu ermöglichen (Bundesgesundheitsministerium, 2020; Kickbusch, 2020). Zudem sollte sich die Bundesregierung dafür einsetzen, zweckmäßige Finanzierung für den Aufbau von Pandemie-Überwachungssystemen zu gewährleisten, um das Risiko für zukünftige Pandemien zu senken. Außerdem sollte sie die industriellen Stärken des Landes ausbauen, sowohl um hochwirksame Impfstoffe zu produzieren und international zu exportieren, als auch um die Resilienz von Wirtschafts- und Lieferketten in der Zukunft zu verbessern.

Einer neuen Bundesregierung bieten sich auch weitere Möglichkeiten für die Zusammenarbeit mit anderen Ländern der Europäischen Union, z. B. bei Initiativen wie der entstehenden Europäischen Gesundheitsunion zur gemeinsamen Erkennung, Vorbereitung und Reaktion auf Gesundheitsbedrohungen.[1] Angesichts der wirtschaftlichen Verluste durch COVID-19, die sich nach Angaben des Internationalen Währungsfonds auf bis zu eine halbe Trillion US-Dollar pro Monat belaufen, ist keine Zeit zu verlieren.

Kurz nach der Bundestagswahl schlagen wir der nächsten Regierung eine Fünf-Punkte-Agenda zur Verbesserung der globalen Gesundheitssicherheit vor, um auf die aktuelle Corona-Pandemie zu reagieren und sich auf künftige Biosicherheitsrisiken vorzubereiten – und damit auch letztlich eine wichtige Grundlage der Globalisierung zu schützen. Und obwohl wir uns auf die deutsche Regierung konzentrieren, können viele der Vorschläge auch im Kontext der Europäischen Union – die nicht im Mittelpunkt dieses Artikels steht – sinnvoll umgesetzt werden.

## Globale Gesundheitssicherheit und Pandemievorsorge

Als Reaktion auf die Corona-Pandemie hat die Bundesregierung ihren Fokus auf die globale Gesundheit und Pandemievorsorge erweitert. Deutschlands neue ressortübergreifende globale Gesundheitsstrategie für 2020 bis 2030, die im Oktober 2020 vom Kabinett verabschiedet

1 Siehe hierzu auch https://ec.europa.eu/info/strategy/priorities-2019-2024/promoting-our-european-way-life/european-health-union_en (27. Oktober 2021).

Abbildung 1

**Zugesagte Beträge im Verhältnis zum Beitragsrichtwert, Top 30 nationale Geldgebende und Europäische Kommission**

in Mrd. US-$

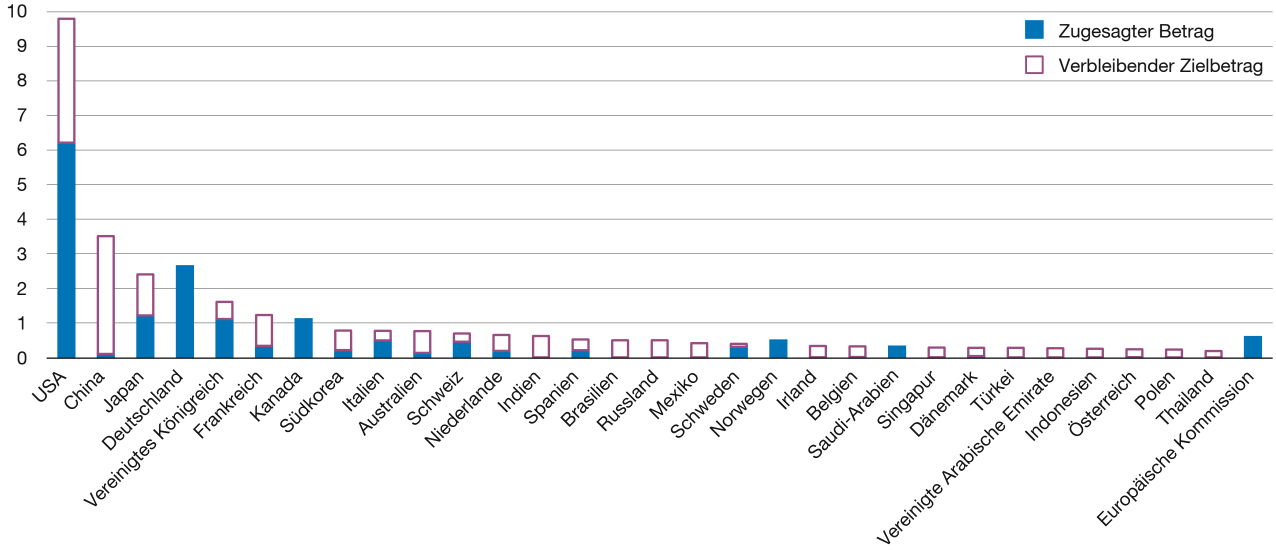

Anmerkung: Das Diagramm zeigt die bis zum 3. September 2021 für ACT-A zugesagten Beiträge im Verhältnis zu den von der WHO berechneten Richtwerten für den „fairen Anteil". Die Zusagen Deutschlands, Kanadas, Norwegens und Saudi-Arabiens übersteigen ihre Zielwerte; für die Europäische Kommission ist kein Zielwert angegeben. Die Berechnung der fairen Anteile orientiert sich an der IWF-Quote und basiert auf dem BIP, der wirtschaftlichen Offenheit und dem Pro-Kopf-BIP, um die Progressivität zu berücksichtigen.

Quelle: WHO Access to COVID-19 tools funding commitment tracker.

wurde, nennt ausdrücklich die Pandemieprävention als oberste Priorität und betont die Stärkung der multilateralen globalen Gesundheitsarchitektur.

Deutschland hat einen großen finanziellen Beitrag zur Reaktion auf die aktuelle Pandemie geleistet. Die Bundesregierung hat mehr als 500 Mio. US-$ für die Corona-Maßnahmen der Weltgesundheitsorganisation bereitgestellt (WHO, 2021a). Um die Entwicklungsländer bei der Bewältigung der Auswirkungen von COVID-19 zu unterstützen, hat Deutschland 10 Mio. Euro für das Health Emergencies Preparedness and Response Trust Fund Programme (HEPR-Programm) bei der Weltbank beigesteuert (WHO, 2021b).

Die deutsche Regierung war auch eine frühe Geldgeberin der Coalition for Epidemic Preparedness Innovations (CEPI) und hat bereits im März 2020 einen ersten Beitrag in Höhe von 140 Mio. Euro geleistet, um die Entwicklung eines COVID-19-Impfstoffs zu beschleunigen (CEPI, 2020), sowie 2,2 Mrd. Euro für den Access to COVID-19 Tools Accelerator (ACT-A) bereitgestellt, von denen der größte Teil zur Unterstützung der Impfstoffplattform COVAX verwendet wird (Auswärtiges Amt, 2021). Darüber hinaus steuert Deutschland über die EU-Finanzierungsmechanismen weitere rund 1 Mrd. Euro bei. So liegt Deutschland bei den finanziellen Beiträgen an zweiter Stelle hinter den USA, die schätzungsweise 6,2 Mrd. US-$ zur Verfügung gestellt haben (vgl. Abbildung 1). Deutschland hat sich außerdem verpflichtet, insgesamt 30 Mio. Impfdosen zu spenden, von denen bereits 6,6 Mio. gespendet worden sind. Die EU hat sich verpflichtet, nun ihrerseits rund 400 Mio. Dosen zu spenden. Darüber hinaus haben die WHO und Deutschland im September 2021 ein neues Zentrum für Pandemie- und Epidemieaufklärung mit Sitz in Berlin eingerichtet, eine globale Plattform zur Vorhersage, Erkennung und Reaktion auf Pandemie- und Epidemierisiken weltweit (WHO, 2021c).

Über die Hilfsmittel hinaus war die Impfstoffproduktion in Deutschland und der EU von entscheidender Bedeutung für die weltweite Versorgung. Bis Anfang August hatten deutsche Unternehmen mehr als 200 Mio. Impfstoffdosen für den Export produziert, Kaufverträge mit 138 Ländern erfüllt und Ausfuhrverbote vermieden. Diese Strategie steht in starkem Gegensatz zu den protektionistischen und nationalistischen Bestrebungen der Regierungen der USA und Indiens im gleichen Zeitraum.

In einer neuen Regierung sollte Deutschland diesen Kurs fortsetzen, indem es die Impfquote im eigenen Land stärker vorantreibt und gleichzeitig gemeinsam mit dem übrigen Europa die Bemühungen beschleunigt, über COVAX mehr überschüssige Impfstoffdosen schneller an Länder mit geringerem Einkommen zu spenden (EEAS, 2020).

Die Finanzierung durch das multilaterale System sollte aufrechterhalten werden. In Deutschland, wie auch anderswo in der Welt, ist eine Abstimmung der Politik und eine Zusammenarbeit zwischen den verschiedenen beteiligten Ministerien sowie den Bundesländern erforderlich, um einen kohärenten und wirkungsvollen Ansatz zur Pandemievorsorge und -bekämpfung zu verfolgen.

### Koordinierung und Finanzierung zur Pandemiebekämpfung

Die G20 und die G7 sind wichtige Foren, um formellen und informellen Gruppenzwang auszuüben. Deutschland kann seine Stimme und Perspektiven nutzen, um andere Industrieländer zum Handeln und zum Engagement in diesen Fragen zu bewegen.

Die G20 gab im Mai 2021 die Erklärung von Rom heraus. In dieser rief sie zu einer verstärkten Koordinierung und zu Investitionen in die langfristige Pandemievorsorge, -prävention, -erkennung und -reaktion, sowie in die Surge Capacity, also die Kapazität medizinischer Systeme im Katastrophenfall auf. Die G7 griff diesen Aufruf im Juni 2021 im Carbis Bay Communiqué auf, versäumte es aber, Wege zur Umsetzung ihrer Vision für die Pandemievorsorge und -reaktion aufzuzeigen. Im Juli 2021 begrüßten die Finanzminister:innen und Zentralbankpräsident:innen der G20 den Bericht des von der G20 beauftragten Hochrangigen Unabhängigen Gremiums zur Finanzierung der globalen Gemeinschaft für Pandemievorsorge und -reaktion (HLIP, 2021).

Jetzt ist es an der Zeit, deutliche Maßnahmen zu ergreifen. Deutschland beteiligt sich aktiv an den Beratungen über die Empfehlungen des Berichts im Rahmen der informellen G20-Finanz- und Gesundheitsgruppe, die im Anschluss an das Juli-Treffen einberufen wurde, um die Empfehlungen des HLIP voranzubringen. Deutschland sollte mit Verbündeten zusammenarbeiten, um eine ehrgeizige und wirksame neue Lenkungs- oder Aufsichtsbehörde und einen multilateralen Finanzierungsmechanismus voranzutreiben, mit dem Ziel, berechenbare, internationale Finanzierung und eine wirksame Verwendung dieser Mittel zu gewährleisten, damit die vorgegebenen Standards für die weltweite Pandemievorsorge und -reaktion eingehalten werden.

Der deutsche G7-Vorsitz im Jahr 2022 bietet auch eine Chance, Engagement für die Pandemievorsorge und -prävention voranzutreiben und einen konkreten Fahrplan für die langfristige Priorisierung der Pandemievorsorge und die entsprechenden institutionellen Regelungen vorzulegen.

### Die Finanzierung globaler öffentlicher Güter in das Kernmandat der Weltbank einbeziehen

Angesichts seiner führenden Rolle in der internationalen Entwicklungsfinanzierung (Platz 2 bei der öffentlichen Entwicklungshilfe der OECD) sollte Deutschland darauf drängen, dass die Finanzierung globaler Kollektivgüter, insbesondere für die Pandemiesicherheit, Teil des Kernmandats der Weltbank und anderer multilateraler Entwicklungsbanken wird.

Die Finanzierung globaler öffentlicher Güter kann als Teil des GRID-Ansatzes (Green, Resilient and Inclusive Development) der Weltbank operationalisiert werden, um langfristige, integrierte und nachhaltige Entwicklungsfortschritte zu erzielen (World Bank Group, 2021a). Die deutsche Regierung kann in Zusammenarbeit mit anderen Ländern und der Weltbank bei der Beratung zu den institutionellen, operativen und finanziellen Regelungen, die für die Umsetzung dieser Veränderungen erforderlich sind, eine wichtige Rolle spielen. Deutschland ist seit langem eine wichtige Stimme für die Finanzierung globaler öffentlicher Güter bei der Reform und Erneuerung der globalen Gesundheits- und Entwicklungsfinanzierungsagenturen und hat die Einrichtung des Pilotprojekts für die Finanzierung globaler öffentlicher Güter bei der Weltbank unterstützt (Birdsall, 2018).

Angesichts der COVID-19-Katastrophe sollte Deutschland zumindest seinen Beitrag zu IDA20 (Fonds zur Bekämpfung von Corona in armen Ländern) bei der Weltbank aufstocken und gleichzeitig die Weltbankleitung auffordern, ein Querschnittsthema zur Krisenvorsorge in IDA20 aufzunehmen (World Bank Group, 2021b). Deutschland könnte auch erwägen, den Zugang zur Vorzugsfinanzierung zu erweitern, um Anreize für die Kreditaufnahme zur Pandemievorsorge zu schaffen, indem es Operationen der International Development Agency (IDA) kofinanziert und die Konditionen der Internationalen Bank für Wiederaufbau und Entwicklung (IBRD) für die Kreditvergabe in Ländern mit mittlerem Einkommen zu diesem Zweck aufkauft. Multilaterale Entwicklungsbanken sind in einzigartiger Weise in der Lage, Anreize für Investitionen in die Pandemieprävention und -vorsorge auf Länder- und Regionalebene zu schaffen und damit ihre bestehende Kreditvergabe zu ergänzen, und Deutschland könnte sich diese Funktionen zunutze machen.

## Finanzielle und politische Unterstützung für die WHO aufrechterhalten

Deutschland ist der wichtigste Geldgeber der WHO. Seine Beiträge zur WHO, einschließlich der bewerteten und freiwilligen Beiträge, machten im Zweijahreszeitraum 2020 bis 2021 fast 12 % aller Mittel aus. Deutschland sollte sich weiterhin mit Nachdruck dafür einsetzen, dass die WHO angemessen und berechenbar finanziert wird und so ihre notwendigen Aufgaben erfüllen kann. Deutschland kann aber auch seine entscheidende Position als einer der wichtigsten Geldgeber nutzen, um die WHO zu mehr Transparenz in ihren Praktiken zu drängen und sich für ein System institutioneller Kontrollen und Gegenkontrollen einzusetzen, das ordnungsgemäß funktioniert. Die Rolle der WHO ist zwar von entscheidender Bedeutung, aber sie ist nur ein Teil des Puzzles.

## Deutschlands industrielle Stärken ausbauen, um Impfstoffe zu produzieren

Deutsche Unternehmen sind heute wichtige Agierende in der Biotechnologie. Dies ist ein Gewinn für Deutschland und den Rest der Welt. Es handelt sich um eine bedeutende wirtschaftliche Chance für Deutschland, aber auch um eine riesige Chance für die Welt. Die neuen Investitionen von BioNTech in die Produktion in Deutschland können nicht nur die Versorgung der EU, sondern der ganzen Welt mit einem hochwirksamen Impfstoff und seinen Wirkstoffen verbessern. Auch wirtschaftlich gesehen ist der Erfolg von BioNTech ein großer Gewinn für die deutsche Wirtschaft. CureVac selbst meldete zwar schlechte Ergebnisse für seinen Impfstoffkandidaten, aber Chad Bown vom Peterson Institute schlägt vor, dass ein Großteil der Lieferkette dieses Unternehmens für die Herstellung eines der anderen mRNA-Impfstoffe umgewidmet werden könnte (Bown, 2021). Deutschland kann bei dieser Umwidmung führend sein. Es gibt auch Möglichkeiten zur Ausweitung des freiwilligen Technologietransfers und von Partnerschaften, um eine verteilte Produktion und die ständige Produktion auf der ganzen Welt zu gewährleisten. Die anfängliche 50-Millionen-Euro-Investition in Südafrika ist ein guter Anfang, aber es kann noch viel mehr getan werden.

Globale Gesundheitssicherheit und -vorsorge sind auch Bereiche für weitere Zusammenarbeit innerhalb der EU, in denen Deutschland eine wichtige Rolle spielen kann. In ihrer Rede zur Lage der Union vom 15. September bekräftigte Kommissionspräsidentin Ursula von der Leyen ihr Engagement für den Aufbau einer Europäischen Gesundheitsunion und schlug eine neue Gesundheitsvorsorge- und Resilienzmission für die gesamte EU vor, die bis 2027 Investitionen in Höhe von 50 Mrd. Euro vorsieht. Die neue deutsche Regierung sollte ihren Teil dazu beitragen.



Literatur

Auswärtiges Amt (2021), Fighting COVID-19 together in a spirit of solidarity: Germany donates vaccines, 29. September, https://www.auswa-ertiges-amt.de/en/aussenpolitik/themen/gesundheit/covax/2396914 (11. Oktober 2021).
Birdsall, N. (2018), On global public goods: it’s not big money but it’s a breakthrough, Center for global development, https://www.cgdev.org/blog/global-public-goods-not-big-money-but-breakthrough (11. Oktober 2021).
Bown, Chad (2021), Don’t let CureVac’s COVID-19 vaccine supply chain go to waste, Peterson Institute for International Economics, 9. August, https://www.piie.com/blogs/realtime-economic-issues-watch/dont-let-curevacs-covid-19-vaccine-supply-chain-go-waste (11. Oktober 2021).
Bundesgesundheitsministerium (2020), Strategie der Bundesregierung zur globalen Gesundheit „Verantwortung — Innovation — Partnerschaft — Globale Gesundheit gemeinsam gestalten“, https://www.bundes-gesundheitsministerium.de/themen/internationale-gesundheitspolitik/global/globale-gesundheitspolitik-gestalten/strategie-der-bundesregierung.html (11. Oktober 2021).
Carbis Bay Communiqué, G7 (2021, Juni), https://www.g7uk.org/wp-content/uploads/2021/06/Carbis-Bay-G7-Summit-Communique-PDF-430KB-25-pages-1-1.pdf (11. Oktober 2021).
CEPI (2020), CEPI gets €140 million funding boost from Germany while expanding coronavirus vaccine search, 12. März, https://cepi.net/news_cepi/cepi-gets-e140-million-funding-boost-from-germany-while-expanding-coronavirus-vaccine-search/ (11. Oktober 2021).
EEAS (2020), Coronavirus: European Union launches „Team Europe“ package to support partner countries with more than €20 billion, Press Release, 8. April, https://eeas.europa.eu/headquarters/head-quarters-homepage/77326/coronavirus-european-union-launches-%E2%80%9Cteam-europe%E2%80%9D-package-support-partner-countries-more-%E2%82%AC20_en (11. Oktober 2021).
HLIP (2021), A global deal for our pandemic age. G20 High Level Independent Panel on Financing the Global Commons for Pandemic Preparedness and Response, Juni, https://pandemic-financing.org/report/foreword/ (11. Oktober 2021).
Kickbusch, I. (2020), Germany’s Role in Global Health, American Institute for Contemporary German Studies, 10. Juni, https://www.aicgs.org/2020/06/germanys-role-in-global-health/ (11. Oktober 2021).
WHO (2021a), Germany Reinforces its commitment to support WHO’s work, 16. Juli, https://www.who.int/news/item/16-07-2021-germany-reinforces-its-commitment-to-support-who-s-work (11. Oktober 2021).
WHO (2021b), Germany contributes €10 Million to WB trust fund for disease preparedness in developing countries, Press release, 2. Februar, https://www.worldbank.org/en/news/press-release/2021/02/02/germany-contributes-10-million-to-wb-trust-fund-for-disease-pre-paredness-in-developing-countries (11. Oktober 2021).
WHO (2021c), WHO, Germany open hub for pandemic and epidemic intelligence in Berlin, 1. September, https://www.who.int/news/item/01-09-2021-who-germany-open-hub-for-pandemic-and-epidemic-intelligence-in-berlin (11. Oktober 2021).
World Bank Group (2021a), From COVID-19 crisis response to resilient recovery — saving lives and livelihoods while supporting green, resilient and inclusive development (GRID), https://thedocs.worldbank.org/en/doc/9385bfef1c330ed6ed972dd9e70d0fb7-0200022021/original/DC2021-0004-Green-Resilient-final.pdf (11. Oktober 2021).
World Bank Group (2021b), IDA20 Replenishment, https://ida.worldbank.org/replenishments/ida20-replenishment (11. Oktober 2021).
